# Exploring Regional Advanced Manufacturing and Its Driving Factors: A Case Study of the Guangdong–Hong Kong–Macao Greater Bay Area

**DOI:** 10.3390/ijerph18115800

**Published:** 2021-05-28

**Authors:** Zixin Dou, Yanming Sun, Tao Wang, Huiyin Wan, Shiqi Fan

**Affiliations:** 1 School of Management, Guangzhou University, Guangzhou 510000, China; 1111965001@e.gzhu.edu.cn; 2 Research Center for High Quality Development of Modern Industry, Guangzhou University, Guangzhou 510000, China; 3 Department of Building Surveying, Faculty of Built Environment, University of Malaya, Kuala Lumpur 50603, Malaysia; 17221416@siswa.um.edu.my; 4 Department of Earth and Environmental Science, University of West Florida, Pensacola, FL 32514, USA; hw34@students.uwf.edu; 5 Department of Engineering, The University of Hong Kong, Hong Kong 999077, China; fsq247@connect.hku.hk

**Keywords:** manufacturing industry, economic factor, environmental factor, technological factor, PPM theory

## Abstract

This study aims to analyze the development trend of the manufacturing industry of the Guangdong–Hong Kong–Macao Greater Bay Area (from 2008 to 2018) by constructing an evaluation system. On the basis of push–pull–mooring theory, we analyze these factors by using an entropy and cluster model. The results show the following: (1) Technological development had an obvious spatial distribution pattern of core regional radiation, while others did not. (2) Economic development was based on the city’s existing industrial development system, while environmental development depended on governmental policies. (3) Compared with the environmental factor, the development trends of the economic and technological factors were more similar. Lastly, we provide four strategies for the development of the manufacturing industry in different cities.

## 1. Introduction

The manufacturing structure of the Guangdong–Hong Kong–Macao Greater Bay Area (GBA) is constantly being upgraded, especially with intelligent manufacturing [1,2], equipment [3], and biomedicine [4]. However, in terms of the “reindustrialization” strategy [5] carried out by the United States, the Sino–United States trade war exposed a lack of key technologies in China’s manufacturing industry, giving it a disadvantage in international competition. Therefore, it is necessary to study the regional advanced manufacturing industry (AMI).

The concept of an AMI has attracted considerable attention from scholars. As an AMI is a high-tech industry with independent intellectual property rights, the concept of an AMI mainly focuses on the technical level, such as system technology [6,7,8,9], information technology [10,11,12,13,14,15], and manufacturing modes [16,17,18]. However, by reviewing the relevant literature, we found that understanding the existing concept of advanced manufacturing involves more technical dimensions, and fewer studies have been conducted from multiple perspectives.

Recent mainstream theories of industrial development are as follows: PEST theory [19,20,21,22,23] aims to grasp the macro environment of the research object as a whole. Through the evaluation of these factors, the future development strategy of the research object is formulated. SWOT theory [24,25,26,27] refers to all factors that are closely related to the research object. System analysis can match these factors and lead to conclusive decision making. Diamond theory [28,29,30,31] judges whether an industry has strong competitiveness by analyzing its overall advantages. However, most existing theories focus on factors that initially affect the industry, ignoring factors that promote or hinder the sustainable development of the industry. Therefore, we used push–pull–mooring (PPM) theory to identify and analyze the sustainable development of the industry.

In recent years, AMI development has become a research hotspot that can mainly be divided into the following three parts: (1) Some scholars have studied manufacturing agglomeration [32]. For example, Mariko [33] reported that the development of emerging industries is a process of dynamic evolution. Martin R. [34] studied the development path of emerging agglomeration. (2) Some scholars have studied green development [35]. For example, Guo Y. et al. [36] found that economic development is positively correlated with the efficiency of green development. Liu S. et al. [37] found that the emission of industrial pollutants is related to industrial agglomeration. Chen D. et al. [38] found that industrial development leads to more carbon dioxide emissions but also reduces the intensity of industrial carbon dioxide emissions. (3) Some scholars have studied innovation ability [39]. For example, Wang L. et al. [40] found that the development of high-tech industries in cities attracts more highly skilled workers and promotes their better development. Wu K. et al. [41] reported that the cultivation of innovation ability is becoming increasingly important as a measure to enhance the sustainability of a regional industry. However, by examining the literature on the development of AMIs, the transformation from traditional to advanced industries from a single perspective is found. There is a lack of systematic analysis of the development of an AMI.

To address these knowledge gaps, (1) on the basis of PPM theory, we defined the regional AMI, which is helpful in expanding the existing advanced manufacturing concept. This involves technological dimension, such as manufacturing technology, and economic and environmental dimensions. (2) On the basis of an entropy and cluster model, the development status of the AMI in regional cities was established. Through horizontal comparative analysis and the vertical historical trend analysis of cities, we analyzed the sustainable development of regional AMI. This provides a guideline for the development of AMIs. Different development patterns for different urban AMI development strategies, such as industrial collaboration, integration, upgrading, and foundation-development strategies, were proposed through the research results. This can help regional cities to reasonably develop their AMIs.

The rest of the paper is arranged as follows: Section 2 summarizes the related literature. Section 3 puts forward the research framework, namely, the research theory, evaluation system, and research method. Section 4 presents the experiments, including data sources and results, as well as the research findings. Section 5 discusses the theoretical and practical implications. Lastly, the conclusion is presented in Section 6.

## 2. Literature Review

### 2.1. AMI Concept

An AMI is a high-tech industry with independent intellectual property rights and is engaged in manufacturing high-value-added products. Recently, the concept of an AMI has experienced an evolution from manufacturing technology to manufacturing mode. (1) In terms of manufacturing technology, to enhance the competitiveness of the manufacturing industry and promote national economic growth, the United States put forward the national advanced manufacturing technology strategic plan, making it a research hotspot. On the one hand, advanced manufacturing technology includes system technology that brings various changes to traditional manufacturing technology, such as microelectronics [6], automation [7], and information technology [8]. On the other hand, it mainly refers to technology that is represented by a new generation of information technology, such as 3D printing [10], artificial intelligence [11], robotics [12], the internet of things [13], and cloud computing [14]. (2) In manufacturing mode, enterprises should cultivate product competitiveness from the perspective of a modular framework [16,17,18]. This both makes the configuration more stable and flexible and enables better commercial applications. For example, Ramadan M. [42] developed an intelligent real-time scheduling module to minimize the manufacturing lead time. Chang J.S. [43] found that the modular design of suppliers affects the flexibility and agility of the supply chain. With the emergence of Industry 4.0 [44], 5G technology [45], the industrial internet of things [46], and new generations of other information technologies, the organizational change strategy based on intelligent empowerment represents the latest mode of advanced manufacturing development, such as the redistribution business [47], ecological marketing manufacturing [48], data-driven manufacturing [49], and customer service manufacturing [50] modes.

### 2.2. Manufacturing Industry Development

The development of AMIs is a research hotspot. The transformation from traditional to advanced industries, such as manufacturing agglomeration, green development, and innovation development, can be examined. (1) The agglomeration of AMIs has new characteristics. Michael Porter defines the emerging industrial agglomeration as a newly established or replastic industrial agglomeration. In terms of manufacturing agglomeration, in 1999, Falck O. [51] evaluated the impact of German agglomeration-oriented economic policy on the innovation activities of high-tech industries. Zheng Q. [52] reported that industrial agglomeration positively impacts the improvement of industrial energy efficiency. Wei W. [53] studied different intensive industries in different regions and found that a high agglomeration level does not always promote the growth of total factor productivity, but a moderate agglomeration level helps to promote economic development. (2) Environmental problems are closely related to the development of the manufacturing industry. In terms of green development, Li L. et al. [54] analyzed the impact of green innovation on the development of highly energy-consuming industrial enterprises in China. (3) In terms of innovation development, Lee Z. et al. [55] reported that the traditional manufacturing industry should focus on design and innovation, which can ensure the development of manufacturing enterprises. Lee H. et al. [56] found that innovative technology affects the productivity of the manufacturing industry. Through the improvement of innovative technology, the development of the manufacturing industry can be guaranteed.

### 2.3. Industry Theory

By reviewing the existing literature, we found theories to explain industrial development and provide a theoretical basis for this study. These theories included the PEST, SWOT, and diamond theories. (1) In terms of PEST theory, Igliński et al. [19] conducted theory analysis on the Polish renewable energy industry. Healey N. [20] conducted PEST analysis of the European transition economy. The results showed that consumer electronics and services are industries with the greatest growth potential. Lu W. et al. [21] used the PEST analysis framework to find the best level of building prefabrication. Zhou S. et al. [22] conducted a risk assessment of distributed wind farm systems on the basis of PEST theory. (2) SWOT theory refers to all kinds of factors that are closely related to the research object. By using system analysis, it can match these factors and produce conclusive decision making. For example, Zima K. et al. [25] used the SWOT analysis method to evaluate the Polish construction industry. Zhao X. et al. [26] used this theory to analyze the development of the shale gas industry in China. Narayan P. [27] used this theory to analyze Fiji’s tourism industry. On this basis, the development strategy of the Xi’an logistics industry was put forward. (3) Diamond theory judges whether an industry has strong competitiveness by analyzing its overall advantages, for example, the renewable energy [29] and manufacturing [31] industries.

These studies have rich theoretical and practical value for analyzing and understanding the impact of industrial development. However, most existing studies focus on factors that directly affect the industry in the short term, often ignoring the situation that promotes or hinders the sustainable development of an industry. To fill this research gap, we used push–pull–mooring (PPM) theory to identify and analyze the sustainable development of an industry.

## 3. Methodology

### 3.1. Research Theory

Push–pull–mooring (PPM) theory originated in the field of demography by capturing influencing factors of people moving from one place to another over a period [57,58]. This theory mainly includes three aspects:

(1) Push factors, such as bad environments and other negative factors from the source;
(2) Pull factors, such as economic development, rich resources, and other positive factors to the destination;
(3) Mooring factors, such as previous experience and ability.

PPM theory was then applied to other fields [59,60], which showed that it has strong expansibility and universality. Significantly, PPM theory explains the process where an industry goes from a bad environment to a better and more sustainable environment. This is in line with the current situation of the development of a regional AMI. Through the application of PPM theory, the sustainable development of a regional AMI can be better guaranteed.

**Figure 1:**
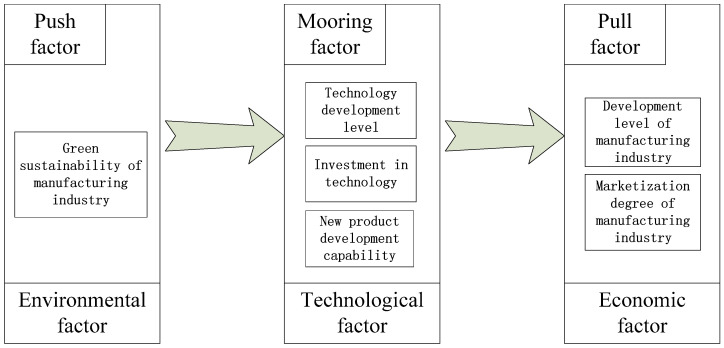
Conceptual framework of a regional advanced manufacturing industry (AMI).

Combined with the relationship between theory and practice, this study investigated the influencing factors of the development of a regional AMI in the framework of PPM theory. The concept of an AMI is shown in Figure 1, where the mooring factor is in the internal development environment of the manufacturing industry. Technological development is inseparable from technological investment and new product design. Only with independent core technology can we effectively drive the intelligent development of an industry. Pull and push factors are in the external development environment of the manufacturing industry, such as economic and environmental factors. The economic effect of industrial agglomeration is to attract the arrival of advantageous industries. If the economic development level of a region is low, it is difficult to attract a large number of manufacturing enterprises to gather and produce a huge trading market. Environmental regulation can force unqualified industries to leave. It is necessary to implement ecological management and control for manufacturing enterprises to form rigid constraints on the ecological environment.

### 3.2. Evaluate System

On the basis of PPM theory, this study classified factors influencing the development of AMI into three aspects. This was used to effectively analyze the development of regional AMI, as shown in Table 1.

Economic factors refer to the degree of the intensive manufacturing industry in the region, which can be reflected in the development level and marketization degree of the manufacturing industry. The X1 improvement can provide an economic guarantee for the manufacturing industry that it first evolves intelligence. X2 reflects the improvement of the marketization degree of the manufacturing industry, which provides power for manufacturing industry development.

The impact of environmental factors, including X3, X4, and X5, on an AMI is lasting and far-reaching. The difficulty is that the atmosphere for advocating green and sustainable development has not been formed, which makes the AMI lack corresponding external supervision. By investigating the waste discharge of the industrial added value per unit scale, we can better understand the degree of industrial pollution.

Technological factors refer to the development level of technology, the ability of new products, and the development potential of technology in a region where an AMI is located. Technological factors refer to the technological development level, input, and output of AMI. X6 and X7 represent the core power to promote manufacturing industry development, which can effectively reflect the level of technological development. X8 and X9 reflect the regional technological input and output, respectively, and—to a certain extent—future technological development potential.

### 3.3. Research Method

By using the entropy method to weigh the Grade 3 index, we evaluated each dimension. The entropy weight method is widely used in other fields [61,62,63], which shows its effectiveness. For the treatment of index standardization, we used a range change method to obtain a standardization matrix $X_{ij}^{\prime }$, and carried out normalization; the formulas are as follows:

(1) $$X_{ij}^{\prime }=\left(X_{ij}-\min X_{ij}\right)/(\max X_{ij}-\min X_{ij})$$

(2) $$X_{ij}^{\prime }=\left(\max X_{ij}-X_{ij}\right)/(\max X_{ij}-\min X_{ij})$$

where i=1, 2,…,m, where *m* is the number of countries; j=1, 2,…,n, where *n* is the number of evaluation indices; $X_{ij}$ represents the *j-*th index value of the *i-*th city. Formula (1) is a positive indicator and Formula (2) is a negative indicator.

Suppose that the evaluation object has *n* samples, where each sample has m indices. $X_{ij}$ represents the original value of the *j*-th index of the ith sample. $X_{ij}^{\prime }$ represents the standard value after processing.

The formula of the weight of the j-th evaluation index is as follows:

(3) $$W_{j}=\left(1-e_{j}\right)/\sum_{j=1}^{n}\left(1-e_{j}\right),$$

where $e_{j}=-k\sum_{j=1}^{n}\left(Y_{ij}-\ln Y_{ij}\right)\geq 0$; $Y_{ij}=X_{ij}^{\prime }/\sum_{i=1}^{m}X_{ij}^{\prime },\;i=1,\;2,\ldots ,m;j=1,\;2,\ldots ,n$*;* and *e* is the entropy value. $Y_{ij}$ is the *i*-th sample value under the *j*-th index.

Thus, the entropy index, $Z_{i}=\sum_{j=1}^{n}W_{j}\times X_{ij}$, of manufacturing competitiveness can be obtained, which is repeated *t* times (*t* = 1, 2, …, *T*), along with the entropy index value of the *t-*th year of the *i-*th city, $Z_{it}$, where $Z_{it,k}=\left\{Z1_{it},Z2_{it},Z3_{it}|k=1,\;2,\;3\right\},$ $Z1_{it}$ is the economic factor, $Z2_{it}$ is the environmental factor, and $Z3_{it}$ is the technological factor.

Second, cluster analysis of dimension scores was undertaken. By maximizing the similarity within a class and minimizing the similarity between classes, data with similar features are clustered. The analytical process is divided into the following steps; the similarity coefficient matrix Q is calculated using the cosine distance formula:

(4) $$\cos\left(\theta_{ik}\right)=\sum_{i=1}^{N}Z_{i,k}Z_{i,k^{\prime }}/\sum_{i=1}^{N}Z_{i,k}{}^{2}\sum_{i=1}^{N}Z_{i,k^{\prime }}{}^{2},k^{\prime }=1,\;2,\;3.$$

By finding the largest element in the similarity matrix, it can select the group classification according to the largest similarity coefficient.

## 4. Results and Analysis

### 4.1. Data Sample and Source

In 2016, the GBA concept first appeared in the outline of the 13th Five-Year Plan. In 2018, the outline of GBA development planning was issued, which indicated that it was necessary to build the strategic mission of the development of an international advanced manufacturing base in the GBA. Therefore, this study mainly focused on the development status of the AMIs in nine cities in the GBA (as shown in Table 2). The data used in this study were from the statistical yearbook of Guangdong Province and the statistical bulletin of the nine cities from 2012 to 2019.

### 4.2. Entropy Results and Analysis

In this study, the entropy analysis method was used to analyze the nine cities from 2011 to 2018. The evaluation scores of the economic, environmental, and technological factors were obtained. To directly reflect the score difference for each city, their scores were mapped to [1,10]. These results are shown in Table 3, Table 4 and Table 5.

**Figure 2:**
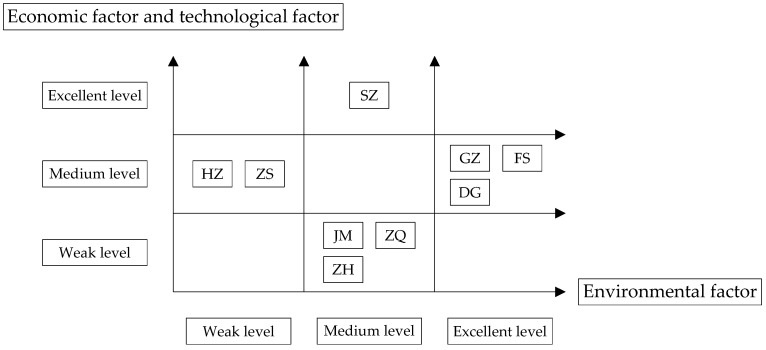
Typical situations of the manufacturing industries in regional cities.

As shown in Table 3, Table 4 and Table 5, the manufacturing industries in different cities have different characteristics; therefore, the development trends of different cities vary and can be divided into four typical situations, as shown in Figure 2.

In Case 1, technology and the economy were excellent and the environment was at a medium level, such as in SZ. The trend of economic development and technology in Shenzhen has always been in first place, which shows that SZ has fully exploited the advantages of the special economic zone. Its economic and technical strength was beyond doubt. Relatively speaking, its environmental development needs further improvement.

In Case 2, technology and the economy were at a medium level and the environment was excellent, such as in GZ, FS, and DG. The economic and technological levels of these cities were still high, and their economic and technological development trend was rela-tively stable. This shows that these cities promoted their economic and technological development through the integration of the information and manufacturing industries. However, their economic and technological levels were far lower than those of SZ. The environmental development trends of these cities were far ahead of those of other cities. This shows that these cities began to manage the pollution of the manufacturing industry after they had found the environmental problems and invested many funds and resources into promoting the sustainable development of the manufacturing industry.

In Case 3, technology and the economy were at a medium level and the environment was weak, such as in HZ and ZS. Although the economic and technological development of HZ and ZS were slow, they were relatively stable. This shows that the development of these cities was insufficient, but they still had potential. However, their green development level was relatively unstable. The backward development of its environment indicates that there was still a large number of traditional industries that needed to be transformed and upgraded.

In Case 4, technology and the economy were weak and the environment was at a medium level, such as in ZH, JM, and ZQ. Although ZH’s economic and technological development were in the third echelon, these two development trends were rising slightly. Its green development trend had also greatly improved, but it was still at the medium level. The economic and technological development levels of JM and ZQ were not ideal. The reason for their environmental level being in the second echelon may have been because they were in a low level of industrialization, which led to fewer pollution sources.

In summary, although different cities placed different emphases on the sustainable development of industry, there were common problems in the development trend of each city. The result of the analysis are as follows: Compared with environmental factors, the development trend of economic and technological factors was similar. For example, the economic and technological development of SZ was excellent, but environmental development still needed to be improved. Environmental factors, however, were not lower than the other two factors. For example, in GZ, FS, and DG, among the three factors, the ranking of environmental development was higher than that of economic and techno-logical factors.

### 4.3. Cluster Results and Analysis

**Figure 3:**
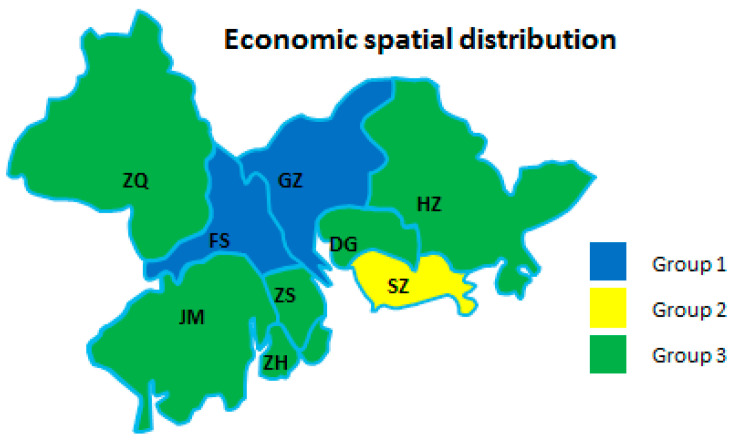
Economic spatial distribution of the nine cities.

**Figure 4:**
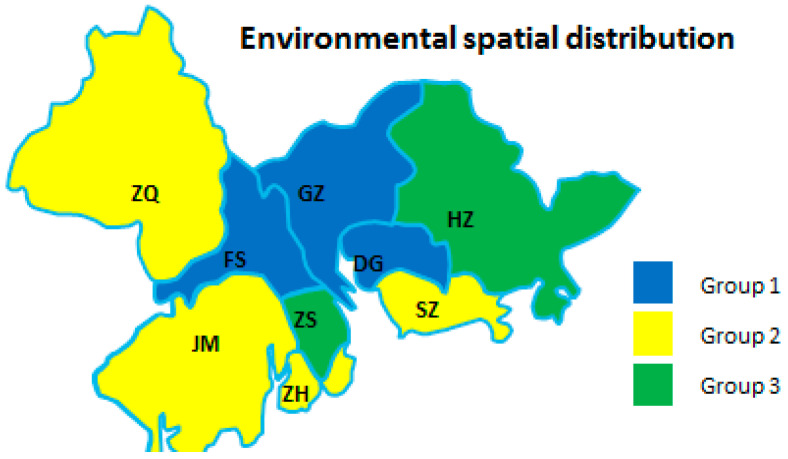
Environmental spatial distribution of the nine cities.

**Figure 5:**
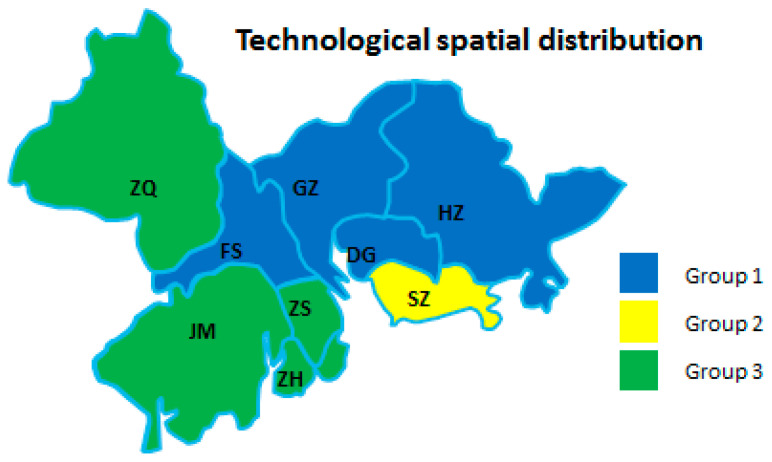
Technological spatial distribution of the nine cities.

From the perspective of each dimension of the manufacturing industry in regional cities, the development degrees of different cities were obviously different. Therefore, it was necessary to divide urban groups using cluster analysis to find commonalities between urban development in different cities. As shown in Table 6 and Figure 3, Figure 4 and Figure 5, the sustainability of a regional AMI has three dimensions: economic, environmental, and technological factors. Due to the different emphases on industrial sustainable development in different cities, the spatial distribution of regional development factors was not the same. The results of the analysis are as follows.

Figure 3 shows that the first-tier city was SZ. Its developed economy and rich market advantages were conducive to the sustainable development of the manufacturing economy. The second-tier cities were GZ and FS. These cities were both economically developed and pioneered industrial internet application areas. Their industrial development scale was at the forefront of the GBA and even the whole country. The rest of the cities belonged to the third tier. The industrial base and business environment of these cities were lower than those of the first and second echelons. To some extent, this restricted the sustainability of the manufacturing economy in these regions.

In summary, the economic development was based on these cities’ existing industrial development system. As a special economic zone, SZ undoubtedly had an economic foundation. However, its radiation effect was not obvious.

Figure 4 shows that GZ, DG, and FS were the top cities in the GBA in terms of environmental factors, and all belonged to the first echelon because these cities have put great effort into the comprehensive treatment of industrial pollution in recent years. This could promote the transformation of the traditional urban–industrial system into a green industrial system, significantly reducing unit pollutant emissions. Unexpectedly, SZ was in the second echelon, perhaps because its industrial output value was too high, leading to a large base of industrial pollution emissions, and relevant green laws and regulations were not keeping up with the development speed of the manufacturing industry. ZH, JM, and ZQ were also in the second echelon. The development of these cities was relatively slow, and the industrial scale was relatively small; therefore, environmental pollution was not serious. HZ and ZS belonged to the third echelon. These cities were in the stage of a developing industrial base, but due to economic scale or lack of technology, they could not effectively control their industrial pollution. Therefore, this restricted the environmental development of the manufacturing industry in these areas.

The regularity of environmental development was not obvious and there was no radiation effect. Combined with the actual situation, environmental development de-pended on the government’s attention, such as issued policies.

Figure 5 shows that these cities could be divided into three echelons. The first-tier city was SZ. Its internet infrastructure was perfect and it had rich scientific and technological resources; the high-tech industry was especially developed. The second-tier cities were GZ, FS, DG, and HZ because of the rich scientific and technological resources in these areas, which provided support for the development of new products and the innovation of new technologies. These cities absorbed the spillover of SZ’s innovation resources and high-end industries. The four other cities were part of the third tier. These cities were far behind the first and second echelons in the development of advanced technology and R&D investment. This greatly limited the technological development of the manufacturing industry in these areas.

In summary, technological development had an obvious spatial distribution pattern of core regional radiation. With SZ as the core, it radiated to neighboring cities.

## 5. Discussion

### 5.1. Theoretical Implication

First, manufacturing sustainability is a hot research topic. However, few people have defined the term “regional AMI.” Therefore, on the basis of PPM theory, we defined the advanced nature of a regional manufacturing industry. On this basis, an evaluation system of three dimensions (economy–environment–technology) was constructed to systematically evaluate a regional AMI. This provided an innovative perspective and enriches the literature on AMIs.

Second, through vertical analysis using a cluster model, and horizontal analysis using the entropy method, this study conducted a more in-depth investigation on the development of regional AMIs, which was helpful for putting forward reasonable suggestions for the sustainable development of AMIs in various cities.

### 5.2. Practical Implication

On the basis of the vertical historical and horizontal comparative analysis of nine cities, we provided four development strategies for a regional AMI according to the three dimensions of the economy, the environment, and technology, which helps regional cities to develop their AMI.

(1) Industrial collaborative development strategy. This is applicable to a city with excellent technological and economic development, while the environmental development of this city needs to be improved, such as in SZ. This city should form a collaborative development of upstream and downstream AMI enterprises and build a complete ecological AMI chain. Through technological and economic advantages, SZ could form an AMI spatial agglomeration to attract more talent and resources. SZ should also strengthen the governance of environmental protection. It can take advantage of technology to reduce industrial pollution emissions, energy consumption, and product waste rates.

(2) Industrial integration development strategy. This is applicable to cities with excellent environmental development while these cities have relatively high technical and economic development potential, such as in GZ, FS, and DG. Through the integration of the manufacturing economy and information technology, these cities could simultaneously improve technology and the economy. First, because of their strong awareness of environmental protection, these cities could prioritize developing a green manufacturing industry and R&D of green technology to ensure the sustainable economic development of their AMI. Second, they should carry out technological innovation to promote the development of industrial intelligence and stimulate economic development. Lastly, from the economic aspect, they should reasonably match market resources and develop new products according to market demand and forecasts of market demand changes.

(3) Industrial upgrading development strategy. This is applicable to cities with medium technological and economic development and weak environmental development, such as in HZ and ZS. These cities should continue to promote the transformation of traditional sunset industries to gain new vitality. Specifically, on the one hand, through economic development, they can resolve the problem of overcapacity and meet the market demand. On the other hand, they can integrate the industry to improve the ability to integrate resources and management efficiency by using information technology. The government should also formulate relevant policies to encourage and support the green energy conservation and emission reduction of the manufacturing industry.

(4) Industrial foundation development strategy. This is applicable to cities with medium environmental development while having significant room for technological and economic development, such as in JM, ZQ, and ZH. On the basis of maintaining the environment, they can lay the foundation for AMI development by attracting foreign investment. The sustainable development of the economy creates an atmosphere of technological development, which can also stimulate economic growth again. Such circular development gives an urban AMI a strong driving force for development.

## 6. Conclusions

There is a lack of in-depth research on the situation that promotes or hinders industrial sustainability. Therefore, this study defined the concept of a regional AMI on the basis of PPM theory, taking nine cities as an example. To understand the potential factors behind AMIs in regional cities, this study also established an evaluation system of influencing factors that can provide a necessary reference for the development mechanism of regional AMIs. The results showed the following: (1) Technological development had an obvious spatial distribution pattern of core regional radiation, while other factors did not. (2) Economic development was based on the city’s existing industrial development system, while environmental development depended on governmental policies. (3) Compared with environmental factors, the development trend of economic and technological factors was more similar. According to these results, we provided four development strategies for regional AMIs, namely, industrial collaboration, integration, upgrading, and industrial foundation development strategies. This is helpful for local governments to formulate appropriate policies and take measures to encourage the sustainable development of an AMI.

Although this study is limited to a specific region, through further research, this knowledge can be globally adopted and verified. For example, different countries have different bases of economic, environmental, and technological development, but most of these factors are common in the development of the global industry. Therefore, this case study was about the GBA, but not limited to it. In terms of the scale of parallel development, it is also very useful for the Tokyo Bay Area in Japan and the San Francisco Bay Area in the United States.

## Figures and Tables

**Table 1:** Evaluation system for a regional AMI.

Grade 1	Grade 2	Grade 3
Economic development	Development level of the manufacturing industry	Industrial added value above a designated size (X1)
	Marketization degree of the manufacturing industry	Total profits of industrial enterprises above a designated size (X2)
Environmental development	Green sustainability of the manufacturing industry	Wastewater discharge of the industrial added value per unit scale (X3)
		Exhaust emissions of the industrial added value per unit scale (X4)
		Solid production discharge of the industrial added value per unit scale (X5)
Technological development	Technological development level of the manufacturing industry	Value added of the advanced manufacturing (X6)
		Value added of the high-tech industry (X7)
	Investment in the manufacturing technology	Internal expenditure on R&D of industrial enterprises above a designated size (X8)
	New product development capability	Output value of new products of industrial enterprises above a designated size (X9)

**Table 2:** Selected cities.

Abbreviation	City	Abbreviation	City	Abbreviation	City
GZ	Guangzhou	FS	Foshan	ZS	Zhongshan
SZ	Shenzhen	HZ	Huizhou	JM	Jiangmen
ZH	Zhuhai	DG	Dongguan	ZQ	Zhaoqing

**Table 3:** Evaluation scores of the economic factor.

		GZ	SZ	ZH	FS	HZ	DG	ZS	JM	ZQ
2011	Score	7.70	10.00	1.09	6.96	1.64	2.66	2.34	2.10	1.00
	Rank	2	1	8	3	7	4	5	6	9
2012	Score	7.46	10.00	1.25	8.22	2.00	3.13	2.34	1.00	1.37
	Rank	3	1	8	2	6	4	5	9	7
2013	Score	8.10	10.00	1.44	8.27	2.14	3.19	1.91	1.00	1.34
	Rank	3	1	7	2	5	4	6	9	8
2014	Score	6.99	10.00	1.42	7.70	1.99	3.08	1.70	1.00	1.21
	Rank	3	1	7	2	5	4	6	9	8
2015	Score	6.36	10.00	1.13	7.30	1.94	2.78	1.53	1.00	1.02
	Rank	3	1	7	2	5	4	6	9	8
2016	Score	6.41	10.00	1.61	7.61	2.36	3.35	1.60	1.15	1.00
	Rank	3	1	6	2	5	4	7	8	9
2017	Score	5.88	10.00	1.90	6.49	2.54	4.14	1.44	1.42	1.00
	Rank	3	1	6	2	5	4	7	8	9
2018	Score	5.99	10.00	1.89	6.37	2.04	3.87	1.41	1.43	1.00
	Rank	3	1	6	2	5	4	8	7	9

**Table 4:** Evaluation scores of the environmental factor.

		GZ	SZ	ZH	FS	HZ	DG	ZS	JM	ZQ
2011	Score	3.21	4.73	1.30	8.85	1.00	10.00	1.33	8.21	4.07
	Rank	6	4	8	2	9	1	7	3	5
2012	Score	6.07	2.56	2.79	10.00	1.00	8.85	4.12	7.65	6.66
	Rank	5	8	7	1	9	2	6	3	4
2013	Score	10.00	4.85	4.64	9.35	4.95	9.92	1.00	6.93	5.27
	Rank	1	7	8	3	6	2	9	4	5
2014	Score	9.87	4.37	5.56	8.98	2.24	10.00	1.00	7.22	5.54
	Rank	2	7	5	3	8	1	9	4	6
2015	Score	9.92	6.27	4.80	6.97	2.36	10.00	1.00	5.66	4.84
	Rank	2	4	7	3	8	1	9	5	6
2016	Score	10.00	4.03	4.85	8.90	1.00	9.56	1.29	3.54	3.94
	Rank	1	5	4	3	9	2	8	7	6
2017	Score	9.97	4.53	4.70	7.71	4.78	10.00	1.00	4.13	4.12
	Rank	2	6	5	3	4	1	9	7	8
2018	Score	10.00	3.65	4.89	8.28	2.82	9.81	1.00	4.13	4.47
	Rank	1	7	4	3	8	2	9	6	5

**Table 5:** Evaluation scores of the technological factor.

		GZ	SZ	ZH	FS	HZ	DG	ZS	JM	ZQ
2011	Score	4.06	10.00	1.46	2.59	2.05	2.28	1.57	1.23	1.00
	Rank	2	1	7	3	5	4	6	8	9
2012	Score	3.92	10.00	1.41	2.75	2.27	2.46	1.60	1.07	1.00
	Rank	2	1	7	3	5	4	6	8	9
2013	Score	3.76	10.00	1.42	2.80	2.45	2.77	1.52	1.06	1.00
	Rank	2	1	7	3	5	4	6	8	9
2014	Score	3.66	10.00	1.40	2.78	2.21	2.84	1.47	1.09	1.00
	Rank	2	1	7	4	5	3	6	8	9
2015	Score	3.59	10.00	1.42	2.69	2.19	2.80	1.46	1.13	1.00
	Rank	2	1	7	4	5	3	6	8	9
2016	Score	3.25	10.00	1.44	2.57	2.12	3.20	1.39	1.14	1.00
	Rank	2	1	6	4	5	3	7	8	9
2017	Score	3.08	10.00	1.52	2.56	2.21	3.53	1.41	1.22	1.00
	Rank	3	1	6	4	5	2	7	8	9
2018	Score	2.97	10.00	1.52	2.51	2.08	3.62	1.33	1.24	1.00
	Rank	3	1	6	4	5	2	7	8	9

**Table 6:** Cluster result of the regional cities.

	GZ	SZ	ZH	FS	HZ	DG	ZS	JM	ZQ
The economic factor	1	2	3	1	3	3	3	3	3
The environmental factor	1	2	2	1	3	1	3	2	2
The technological factor	1	2	3	1	1	1	3	3	3

## Data Availability

The data in this paper are from the statistics bureau of Guangdong province. http://stats.gd.gov.cn/.
